# Kinetic Characterization and Effect of Immobilized Thermostable *β*-Glucosidase in Alginate Gel Beads on Sugarcane Juice

**DOI:** 10.1155/2014/178498

**Published:** 2014-02-20

**Authors:** Keerti, Anuradha Gupta, Vinod Kumar, Ashutosh Dubey, A. K. Verma

**Affiliations:** ^1^Department of Biochemistry, College of Basic Sciences and Humanities, G. B. Pant University of Agriculture and Technology, Pantnagar 263145, India; ^2^Akal School of Biotechnology, Eternal University, Baru Sahib, Sirmour 173101, India

## Abstract

A thermostable *β*-glucosidase was effectively immobilized on alginate by the method of gel entrapment. After optimization of immobilized conditions, recovered enzyme activity was 60%. Optimum pH, temperature, kinetic parameters, thermal and pH stability, reusability, and storage stability were investigated. The *K*_*m*_ and *V*_max_ for immobilized *β*-glucosidase were estimated to be 5.0 mM and 0.64 U/ml, respectively. When comparing, free and immobilized enzyme, change was observed in optimum pH and temperature from 5.0 to 6.0 and 60°C to 80°C, respectively. Immobilized enzyme showed an increase in pH stability over the studied pH range (3.0–10.0) and stability at temperature up to 80°C. The storage stability and reusability of the immobilized *β*-glucosidase were improved significantly, with 12.09% activity retention at 30°C after being stored for 25 d and 17.85% residual activity after being repeatedly used for 4 times. The effect of both free and immobilized *β*-glucosidase enzyme on physicochemical properties of sugarcane juice was also analyzed.

## 1. Introduction

*β*-Glucosidase (*β*-D-glucoside glucohydrolase; EC 3.2.1.21) is a part of multienzyme cellulase complex, whose synthesis and action are intricately controlled by regulatory mechanisms in the organisms that produce these enzymes. The enzymatic hydrolysis of cellulose involves three types of cellulase activities (cellobiohydrolases, endoglucanases, and *β*-glucosidases) working in synergy [1, 2].

*β*-Glucosidases hydrolyze *β*-D-glycosidic bond to release nonreducing *β*-D-glucose residue and terminal aglycone. These are widely used in the various biotechnological processes including aroma and flavour enrichment [3], discoloration of fruit juices prevention [4], and organoleptic properties of citrus fruit juices improvement, in which the bitterness is in part due to a glucosidic compound, naringin (4,5,7-trihydroxyflavanone-7-rhamnoglucoside) [5]. *β*-Glucosidase also acts as a key enzyme in the enzymatic release of aromatic compounds from glucosidic precursors present in fruits and fermentation products [6]. Transglycosylation reactions by *β*-glucosidase have great importance in wine or beverage industry because of their abilities to improve the aroma [7]. The synthetic activity of *β*-glucosidase can be used in the preparation of a variety of compounds such as oligosaccharides and glycoconjugates that have potential for use as agrochemicals and drugs. *β*-Glucosidase, produced intracellularly by many microorganisms, usually shows a broad specificity and also transferase activity [8].

Nevertheless, the applications of enzyme in industry remain limited due to the high production cost, stability, and need for repeated enzyme purification. The main strategy to increase the enzyme stability and reusability is the immobilization of enzyme. Some of the more significant advantages of immobilized enzymes over their soluble counterparts include the enhanced stability under extreme conditions of temperature, pH, and organic solvents; recovery; and subsequent applicability to continuous processes [9].

Alginate, a polysaccharide consisting of glucuronic acid and mannuronic acid moieties, has been found to be a matrix of priority due to its biocompatibility and processivity [10]. It is a reversibly soluble insoluble polymer which changes solubility in the presence of calcium [11]. To date, cross-linked alginate has been successfully used for encapsulation of many biological molecules [12, 13].

The present study describes the immobilization of *β*-glucosidase in alginate gel beads and the effect of this immobilization on kinetic characteristics of immobilized *β*-glucosidase in comparison with free enzyme. For application purposes, the effect of both free and immobilized *β*-glucosidase enzymes on physicochemical properties of sugarcane juice was also analyzed.

## 2. Material and Methods

### 2.1. Chemicals and Bacterial Culture

All chemicals, media, and components used were of analytical grade and obtained from Sigma Chemicals Ltd., Himedia Laboratories Ltd., GeNei, SRL, and Merck Pvt. Ltd. Recombinant *β*-glucosidase from *Bacillus subtilis* strain PS (identified using 16S rDNA sequencing; GenBank Accession number JQ066263) cloned in *E. coli* DH5*α* in our laboratory was used in this study [14]. The cloned *β*-glucosidase produces extracellularly by the bacterial cell.

### 2.2. Production and Partial Purification of *β*-Glucosidase

*E. coli* DH5*α* containing recombinant enzyme was cultured in Luria broth in 250 mL conical flasks using incubator shaker (150 rpm) at 37°C for 12 h. For extraction of extracellular *β*-glucosidase enzyme, cells were harvested at 10,000 rpm for 30 minutes at 4°C and supernatant was collected and assayed for *β*-glucosidase activity. *β*-Glucosidase enzyme was purified partially using ammonium sulfate fractionation followed by dialysis. All the purification steps were performed at 4°C. Crude enzyme in the cell free supernatant was precipitated by adding ammonium sulfate up to 70%. The precipitates were separated by centrifugation and resuspended in acetate buffer (100 mM, pH 5.0) and dialyzed against the same buffer overnight with two buffer changes. The *β*-glucosidase activity after dialysis was measured and it was used for further studies.

### 2.3. Immobilization of *β*-Glucosidase in Ca-Alginate Gel Beads

Calcium alginate gel beads were prepared as described by Busto et al. [15]. Briefly, sodium alginate solution, 1%, 2%, 3%, 4%, and 5%, was prepared in a suitable amount of enzyme. This solution was dropped in 0.05 M, 0.1 M, and 0.2 M calcium chloride solution under continuous stirring. The beads were cured for 1–5 hrs in the calcium chloride solution, washed several times with a 0.03 M CaCl_2_ solution until no enzyme activity was observed in the final washing, and stored at 4°C in this solution.

### 2.4. Determination of *β*-Glucosidase Enzyme Activity

*β*-Glucosidase activity was evaluated spectrophotometrically using pNPG as an artificial substrate. The reaction mixture, containing 100 *μ*L *β*-glucosidase enzyme extract in acetate buffer (pH 5.0, 100 mM) and 100 *μ*L of pNPG in similar buffer, was incubated for 30 min at 60°C. The reaction was stopped by adding 2 mL of 1 M Na_2_CO_3_ solution and the absorbance was measured at *λ*_405_ nm [16]. The activity of immobilized *β*-glucosidase was determined using the procedure given above except 0.2 g immobilized enzyme was used in place of 100 *μ*L enzyme extract.

### 2.5. Kinetic Characterization of Immobilized and Free Enzyme

The kinetic constants of Michaelis values (*K*_*m*_ and *V*_max⁡_) for the free and immobilized enzyme preparations were determined using Lineweaver-Burk plot by measuring the enzymatic activity at different substrate concentrations (1–15 mM). The turnover number and catalytic efficiency were also determined.

### 2.6. Effect of pH and Temperature on Free and Immobilized *β*-Glucosidase Activity

The optimum pH for *β*-glucosidase activity was studied over a pH range of 3 to 10 to determine the activity of free as well as immobilized enzyme. Citrate buffer (pH 3–6), phosphate buffer (pH 7-8), and glycine-NaOH buffer (pH 9-10) were used to determine enzyme activity. The optimum temperature for *β*-glucosidase activity was determined by incubating the reaction mixture over the temperature range of 40–80°C at the optimum pH.

### 2.7. Effect of pH and Temperature on Stability of Extracellular Free *β*-Glucosidase

The stability of the enzyme was determined by preincubating the enzyme for 30 min at 37°C with various buffers having a pH range of 3 to 10 as mentioned earlier. After incubation, the residual enzyme activity (%) was measured using acetate buffer (pH 5.0) as explained earlier. The thermal stability of the enzyme was studied by preincubating the enzyme at different temperatures ranging from 40 to 80°C for 0–120 min at optimum pH.

### 2.8. Storage Stability and Reusability of Immobilized *β*-Glucosidase

The storage stability of immobilized *β*-glucosidase at 4°C and 30°C was measured by calculating the residual activity at the interval of 5 d up to 30 d. Reusability of immobilized enzyme was also investigated by measuring its activity after repeated cycles of use.

### 2.9. Estimation of Reducing Sugar in Sugarcane Juice

Sugarcane juice was incubated at 60°C for 30 min in the presence of free and immobilized *β*-glucosidase in alginate gel and the reducing sugar was estimated by Somogyi's method [17]. Determination of relative density and viscosity coefficient of treated sugarcane juice with free and immobilized enzyme was done by density bottle method and Ostwald's viscometer.

### 2.10. Absorption Spectra of Sugarcane Juice

Sugarcane juice was incubated for 30 min at 60°C in the presence of free and immobilized *β*-glucosidase and then the samples were diluted to five times with distilled water. The absorption spectra of untreated and treated juice were analyzed in visible range (400–700 nm).

### 2.11. Statistical Analysis

The mean values and standard deviation of three experiments were calculated and presented on the figures as error bars. One-way ANOVA at the significance levels of 0.005 and 0.001 was performed using Microsoft excel 2007 statistical tools.

## 3. Results and Discussion

### 3.1. Immobilization of *β*-Glucosidase in Gel

**Figure 1:**
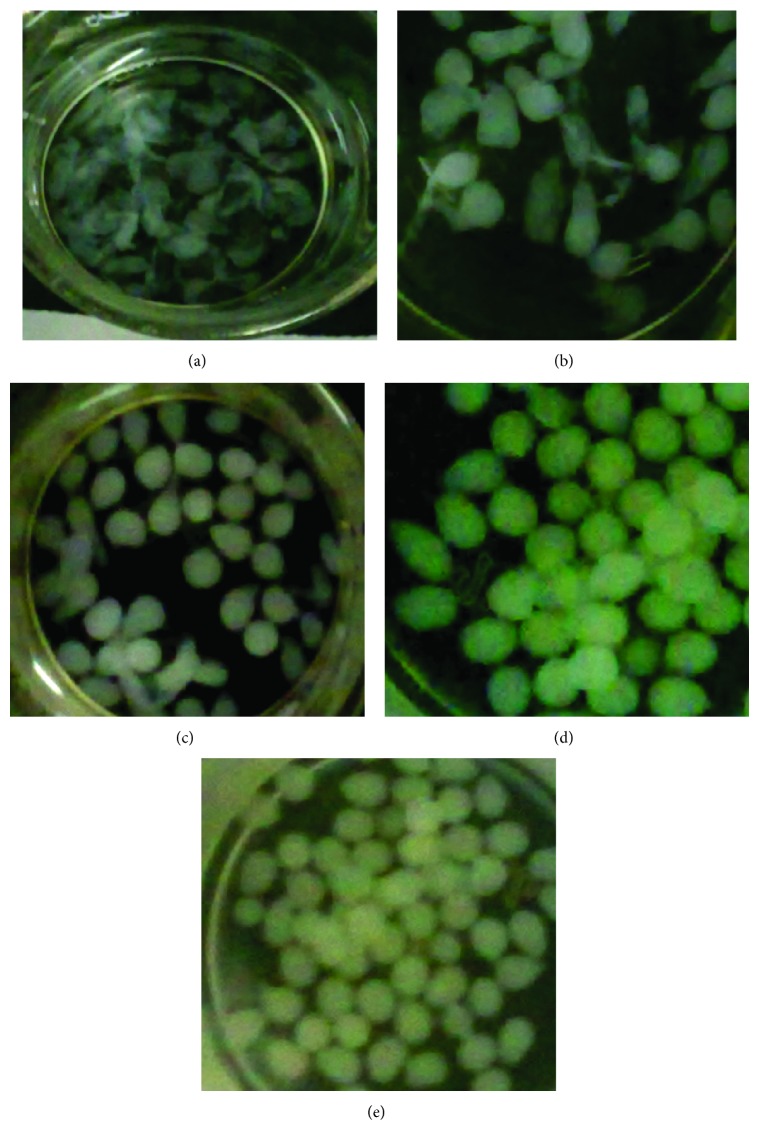
Immobilization of *β*-glucosidase in different concentrations of sodium alginate ((a) 1% sodium alginate; (b) 2% sodium alginate; (c) 3% sodium alginate; (d) 4% sodium alginate; (e) 5% sodium alginate).

The effects of Na-alginate and CaCl_2_ concentrations on the bead formation and immobilization of enzyme revealed that maximum immobilization efficiency (60% *β*-glucosidase activity) was obtained with 3% sodium alginate and 0.2 M CaCl_2_ for 1 h (Figure 1). A similar high level of activity was obtained by Ortega et al. [18], where *β*-glucosidase from *Aspergillus niger* was immobilized in calcium alginate, using an alginate concentration of 3%. No significant effect was observed on the *β*-glucosidase immobilization with various concentrations of the immobilization time. On increasing CaCl_2_ concentration from 0.05 to 0.2 M, the stability of the gel increased without loss of enzyme activity, which was in agreement with the results obtained by Jain and Ghose [19]. Lower immobilization efficiency of *β*-glucosidase at sodium alginate concentrations below 3% was suggested to be due to larger pore sizes of the less tightly cross-linked gels [20], and at sodium alginate concentrations above 3% might be due to lack of uniform pore size because of high viscosity of the enzyme alginate mixture.

### 3.2. Kinetic Characteristics of Free and Immobilized *β*-Glucosidase

The effect of the substrate concentration on the rate catalyzed by free and immobilized enzyme was studied using varying concentrations (1–15 mM) of pNPG as the substrate. Michaelis constant (*K*_*m*_) and the maximum reaction velocity (*V*_max⁡_) of free and immobilized enzyme were calculated from the Lineweaver-Burk plot. The immobilized enzyme in alginate showed an apparent *K*_*m*_ value (*K*_*m*_ = 5.0 mM) higher than the free enzyme (*K*_*m*_ = 3.6 mM). Similar results were reported by Quiroga et al. [21] for immobilized araujiain, a cysteine phytoprotease in calcium alginate gel beads. An increase in *K*_*m*_ after immobilization indicates that the immobilized enzymes have an apparent lower affinity for their substrate than the free enzyme. This may be caused by the support steric hindrance of the active site, by the loss of enzyme flexibility necessary for substrate binding, or by diffusional resistance to substrate transport [22]. In addition, a decrease in *V*_max⁡_ was observed for immobilized enzyme (0.745 *μ*mol min^−1^ mL^−1^) as compared to free enzyme (0.94 *μ*mol min^−1^ mL^−1^). This decrease might be attributed to limited accessibility of substrate molecules to the active sites of the enzyme and the interaction of the enzymes with the functional groups on the surface of beads or large areas of contact between enzyme and support. The apparent *K*_cat_ values of free and immobilized *β*-glucosidase under standard assay conditions were 3.13 × 10^−4^ s^−1^ and 4.15 × 10^−4^ s^−1^, respectively. The catalytic efficiency of free and immobilized *β*-glucosidase was 0.87 × 10^−4^ and 0.83 × 10^−4^, respectively.

### 3.3. Effect of Temperature on Free and Immobilized *β*-Glucosidase

**Figure 2:**
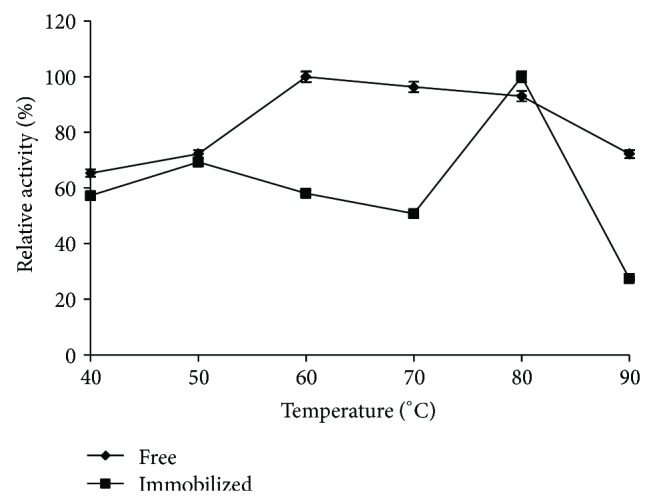
Relative activity (%) of free and immobilized *β*-glucosidase at different temperatures.

Effect of temperature variations on free and immobilized enzyme activity was investigated. Reactions were carried out at pH 6.0 and temperature influence was studied within the 40–90°C range (Figure 2). The optimum temperature of the free enzyme was 60°C but after the entrapment process a shift in such temperature was observed and the immobilized enzyme exhibited the highest activity at 80°C in gel, since hydrophobic and other secondary interactions of the immobilized enzyme might impair conformational flexibility needing higher temperatures for the enzyme molecule to recognize and attain a proper conformation in order to keep its reactivity [23]. Thereafter, a loss in activity above 80°C might be due to the denaturation of some enzyme molecules, leaching of enzyme from the swollen polymer matrix, and degradation of polymer matrix [24]. Further, the high activity of immobilized enzyme at 50°C probably is a result of favoured adsorption of enzymes [25]. Lower activity of the immobilized enzyme has been observed during these assays as compared to the free enzyme. It might be due to decreased affinity of the enzyme for the substrate caused by internal diffusion of the immobilized enzyme [26].

The dependence of the rate constant on temperature of an enzyme catalyzed reaction can be represented by the Arrhenius equation [27]. For many reactions, the *E*_*a*_ values are in the range of 50–100 KJ mol^−1^. But in the case of enzyme catalyzed reactions, the *E*_*a*_ values are generally lower than those of non-enzyme-catalyzed reactions. The observed activation energies for free and immobilized enzyme were 54 and 14.44 KJ mol^−1^, respectively. Similar results were obtained in case of aminoacylases immobilized by alkylation with iodoacetyl cellulose [28]. The values of *E*_*a*_ for the immobilized enzyme are smaller than that for the free enzyme, implying that the immobilized enzymes are less sensitive [29].

**Figure 3:**
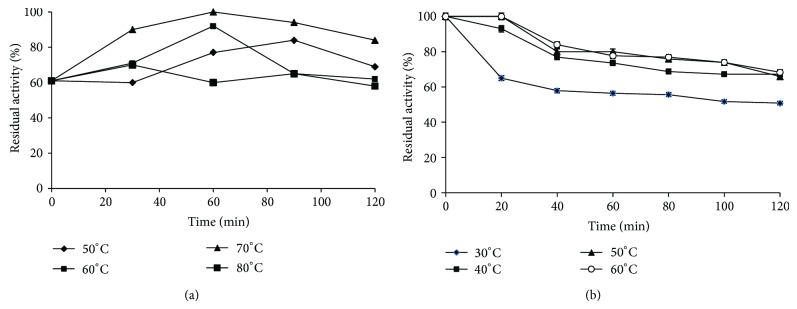
Residual relative activity (%) of (a) immobilized and (b) free *β*-glucosidase to determine its stability at different temperatures.

The rates of thermal inactivation of the free and immobilized *β*-glucosidase were studied in the temperature range of 30–60°C and 50–80°C for 0–120 min (Figures 3(a) and 3(b)). The results indicated that the free *β*-glucosidase was fairly stable at the temperature range from 50 to 60°C while the immobilized *β*-glucosidase was fairly stable at 60–80°C. The above results suggest that the alginate matrix preserved the structure of the enzyme after immobilization process and it protected the enzyme from conformational changes caused by effects of temperature. The activity of immobilized enzyme decreased slowly and still retained 58% of its residual activity at 80°C. Similar results were obtained in immobilized araujiain without any significant loss in its activity at the studied temperatures [21]. The thermal stability of immobilized *β*-glucosidase increased considerably as a result of immobilization in the sodium alginate beads. This is because of immobilization and cross-linking which provided a more rigid external backbone for enzyme molecules. As a result, the effect of higher temperature in breaking the interactions that were responsible for the proper globular, catalytic active structure became less prominent, thus increasing the thermal stability of the immobilized enzyme [30].

### 3.4. Effect of pH on Free and Immobilized *β*-Glucosidase

**Figure 4:**
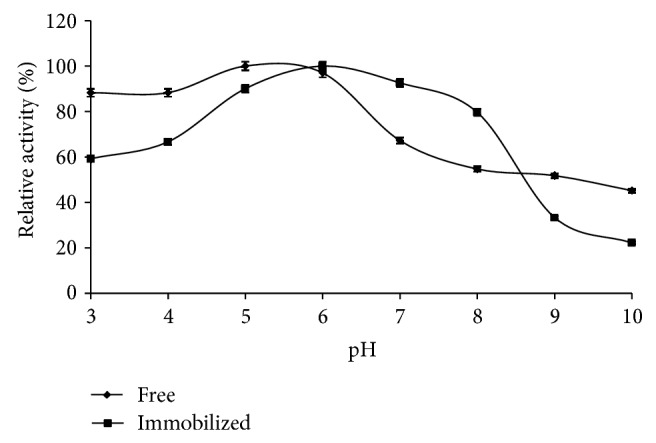
Effect of pH on the relative activity of free and immobilized *β*-glucosidase.

The optimum pH of free and immobilized *β*-glucosidase was studied at various pH values (3.0–10.0) (Figure 4). The results indicated that the optimum pH values of free and immobilized *β*-glucosidase were 5.0 and 6.0, respectively. The pH shifts towards alkaline value upon immobilization are suggested to be because of secondary interactions between the enzyme and the polymeric matrix [31, 32]. The maximum activity range of immobilized enzyme over the pH 6.0–8.0 revealed its resistance to the alkaline changes in medium as compared to the free enzyme [21].

**Figure 5:**
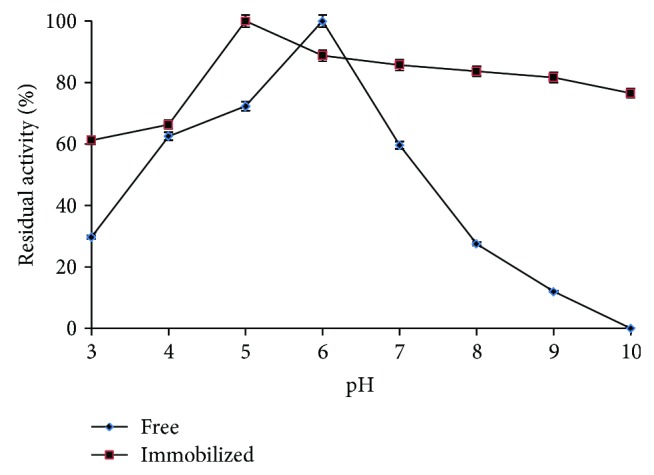
Residual relative activity (%) of free and immobilized *β*-glucosidase to determine the stability at different pH values.

The pH stabilities of free and immobilized *β*-glucosidase are compared at different pH values (3.0–10.0) after incubation for 30 min at 37°C (Figure 5). The results showed that immobilized *β*-glucosidase in alginate gel had the highest stability around pH value of 5.0, whereas the free *β*-glucosidase had the highest stability around pH 6.0. The activity of immobilized *β*-glucosidase was higher than that of free *β*-glucosidase at pH < 4.0 or pH > 7.0, suggesting that immobilization seems to confer some kind of protection to the enzyme when the reaction media presented a pH value at the scope of acidity or alkalescence [30]. Similar results were observed in case of immobilized laccase, where on magnetic chitosan microspheres it was stable in the pH range 5.0–6.0 while free laccase was stable in the pH range 7.0–9.0 [33]. This indicates that the immobilization appreciably improves the stability of laccase in the acidic region. Therefore, entrapped enzyme displays greater pH stability at optimum pH and exhibits a better stability in neutral and basic medium than free enzyme.

### 3.5. Storage Stability and Reusability of Immobilized *β*-Glucosidase

**Figure 6:**
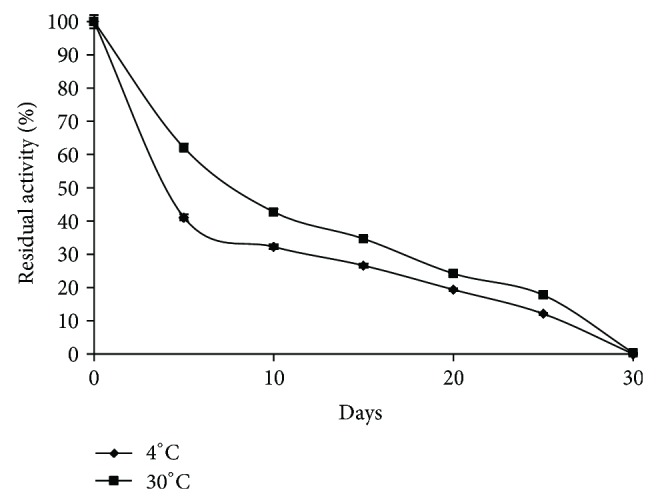
Activity retention (%) of immobilized *β*-glucosidase at 4°C and 30°C.

Storage stability for the immobilized enzyme was one of the significant indexes to evaluate the properties of enzyme, which can make the immobilized enzyme more advantageous than that of the free one. In general, if an enzyme is in solution, it is not stable during storage, and the activity is gradually reduced [34]. The immobilized enzyme in alginate was stored for 30 d at 4°C and 30°C. The residual activity was determined as a function of time (Figure 6). The residual activity of free *β*-glucosidase was found gradually decreasing to 3.05% from 0 to 4th d at 4°C and no residual enzyme activity was observed after 1 d when the enzyme was stored at 30°C. However, the immobilized *β*-glucosidase in alginate retained about 17.74% and 12.09% of its original activity at 4°C and 30°C after 25 d, respectively. This extended stability could be attributed to the prevention of structural denaturation as a result of the immobilization of *β*-glucosidase. Yahşi et al. [35] reported that hydrogel carriers such as poly (acrylamide-co-acrylic acid) and Ca-alginate provide a protective microenvironment for enzymes and yield higher stabilities.

The reuse number of immobilized enzymes is one of the most important aspects for industrial application. An increased stability could make the immobilized enzyme more advantageous than the free form [30]. The immobilized *β*-glucosidase in alginate was reused for 4 times and the residual activity gradually decreased to 17.85%. Thus the immobilized enzyme activities decreased while reused number increased. These results could be explained by the inactivation of enzyme caused by the denaturation and the leakage of enzyme from gels upon use and diffusional effects [36].

### 3.6. Treatment of Sugarcane Juice

**Figure 7:**
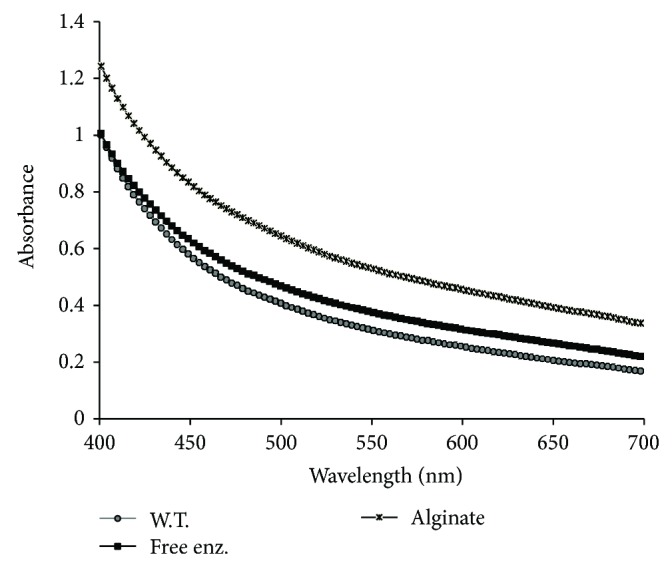
Absorption spectra of treated and untreated sugarcane juice by free and immobilized *β*-glucosidase (W.T.: juice without treatment, free enz.: juice treated with free *β*-glucosidase, and alginate: juice treated with immobilized *β*-glucosidase in alginate beads).

The effect of both free and immobilized *β*-glucosidase enzyme on sugarcane juice was analyzed. It was observed that after sugarcane juice was treated with free and immobilized *β*-glucosidase for 30 min at 60°C, the physicochemical properties of juice changed, such that viscosity of juice was decreased from 2.009 centipoise of untreated to 1.350 centipoise of treated with free *β*-glucosidase and 1.499 centipoise of treated with immobilized *β*-glucosidase. The reducing sugar was increased from 6.348 g/L in untreated juice to 9.438 g/L in free *β*-glucosidase and 8.134 g/L in immobilized *β*-glucosidase (Table 1). While the absorption spectra of treated juice showed increased absorbance in the visible region (400–700 nm) (Figure 7), the increase of absorption spectra (hyperchromic shift) showed that the *β*-glucosidase hydrolyzed the glycosidic linkage between sugar and phenolic compounds present in the sugarcane juice. When these phenolic compounds are released, the sugarcane juice appears dark in colour. These glucosidic compounds play an important role in growth and reproduction, providing protection against pathogens and predators [37], besides contributing towards the colour and sensory characteristics of fruits and vegetables [38].

## 4. Conclusion

The *β*-glucosidase enzyme was successfully immobilized with the best immobilization efficiency obtained in 3% sodium alginate in 0.2 M CaCl_2_ for 1 h of curing. The *K*_*m*_ value of immobilized enzyme was higher than that of the free one which may be due to the decreased affinity of enzyme towards the substrate, whereas the *V*_max⁡_ value of immobilized enzyme was lower than that of the free one which may be due to less accessibility of substrate to the enzyme. The immobilized enzyme exhibited a shift in optimum temperature from 60°C to 80°C which indicates that the immobilization process improves the activity and stability of *β*-glucosidase enzyme. The increased reusability, higher pH and storage stability of immobilized enzyme as compared to the free enzyme will be important for its sustained use and economic viability of biosynthetic processes.

## Figures and Tables

**Table 1:** Effect of free and immobilized enzyme on physicochemical properties of sugarcane juice.

Type of juice	Reducing sugar (g/L)	Density (g/mL)	Viscosity (centipoise)
Untreated juice	6.348	1.065	2.009
Treated with free *β*-glucosidase	9.438	1.066	1.351
Treated with immobilized *β*-glucosidase	8.134	1.06	1.449
